# Multidimensional Patient-Reported Problems within Two Weeks of HIV Diagnosis in East Africa: A Multicentre Observational Study

**DOI:** 10.1371/journal.pone.0057203

**Published:** 2013-02-19

**Authors:** Victoria Simms, Nancy Gikaara, Grace Munene, Mackuline Atieno, Jeniffer Kataike, Clare Nsubuga, Geoffrey Banga, Eve Namisango, Suzanne Penfold, Peter Fayers, Richard A. Powell, Irene J. Higginson, Richard Harding

**Affiliations:** 1 London School of Hygiene and Tropical Medicine, London, United Kingdom; 2 Cicely Saunders Institute, King's College London, London, United Kingdom; 3 African Palliative Care Association, Kampala, Uganda; 4 Department of Population Health, University of Aberdeen, Aberdeen, United Kingdom; Indiana University, United States of America

## Abstract

**Objectives:**

We aimed to determine for the first time the prevalence and severity of multidimensional problems in a population newly diagnosed with HIV at outpatient clinics in Africa.

**Methods:**

Recently diagnosed patients (within previous 14 days) were consecutively recruited at 11 HIV clinics in Kenya and Uganda. Participants completed a validated questionnaire, the African Palliative Outcome Scale (POS), with three underpinning factors. Ordinal logistic regression was used to evaluate risk factors for prevalence and severity of physical, psychological, interpersonal and existential problems.

**Results:**

There were 438 participants (62% female, 30% with restricted physical function). The most prevalent problems were lack of help and advice (47% reported none in the previous 3 days) and difficulty sharing feelings. Patients with limited physical function reported more physical/psychological (OR = 3.22) and existential problems (OR = 1.54) but fewer interpersonal problems (OR = 0.50). All outcomes were independent of CD4 count or ART eligibility.

**Conclusions:**

Patients at all disease stages report widespread and burdensome multidimensional problems at HIV diagnosis. Newly diagnosed patients should receive assessment and care for these problems. Effective management of problems at diagnosis may help to remove barriers to retention in care.

## Introduction

A very high proportion of people newly diagnosed with HIV lose contact with health care services and do not initiate antiretroviral therapy (ART) at the most appropriate time, leading to increased mortality and morbidity[1]. Improved linkage of services and retention in care is a priority in order to optimise treatment outcomes[2]. Correct management of patients at diagnosis is the gateway to other health services including ART. Unaddressed social, emotional and informational needs at the time of HIV diagnosis are thought to be a cause of avoidant coping strategies such as denial[3]. Depressive symptoms are very common for HIV positive people presenting for a test, and impact upon CD4 test uptake[4]. Common physical and psychological symptoms (pain, vomiting, fatigue, confusion and hopelessness) are reported as impediments to ART adherence[5], [6]. A multidimensional approach to patient assessment at diagnosis which incorporates physical, psychological, emotional, social and informational elements would enable early management of these problems, which could help to remove some of the barriers that prevent patients from remaining in care[4].

From an early stage[7], HIV has a multidimensional impact on patient wellbeing, with negative physical[8], [9], psychological[10], [11], social[12], [13] and spiritual[14] repercussions, but very little is known about patient-reported experience at the specific time of HIV diagnosis[15]. Retrospective research is the most common method, but is unsuitable due to severe bias in the recall of emotionally significant events[16]. An alternative option is to measure outcomes just prior to diagnosis in patients later identified as HIV positive[4], but this approach, while suitable for research, is not realistic in terms of clinical practice.

A systematic review of multidimensional problems reported by newly diagnosed HIV patients[15] found only two prospective studies that recruited an outpatient sample within two weeks after diagnosis in a resource-poor setting (typifying the majority of new HIV diagnoses[17]), and both had methodological flaws. One was a comparative study of military personnel in Nigeria which identified lowered physical, mental, role and social functions associated with HIV diagnosis using the MOS-HIV questionnaire, but did not represent the wider population[18]. The other, a cross-sectional survey in India, found high prevalence of pain, physical symptoms, and emotional, spiritual and work-related problems, but used convenience sampling and an unvalidated questionnaire[19].

This study aimed to measure prevalence, severity and risk factors for multidimensional problems in the first two weeks after HIV diagnosis in a sample of outpatients in sub-Saharan Africa.

## Methods

### Ethics statement

Ethical approval was obtained from Ugandan National Council for Science and Technology (UNCST, Ref. SS 1964), the Kenyan Medical Research Institute (Ref. KEMRI/RES/7/3/1) and the College Research Ethics Committee at King's College London (Ref. CREC/06/07-140). All participants gave informed written consent prior to data collection.

### Study design

The data were collected as part of a previously described mixed-methods evaluation of care and support[20]. Patients presenting with a new HIV diagnosis at eleven HIV comprehensive care clinics attached to urban district or national hospitals in Kenya and Uganda were recruited consecutively in 2008. The clinics are described in detail in two evaluation reports[21], [22]. In Kenya two clinics were in the west, three in central Kenya and one in Mombasa. In Uganda, three clinics were in Kampala and two in southern towns. The total length of recruitment time at all clinics was 819 working days. All eligible patients were approached for consent. Eligibility criteria were: self-report that their first positive HIV diagnosis had occurred within the previous 14 days, aged 18 or over, not requiring hospital admission. Participants were interviewed four times at monthly intervals. Cross-sectional data from the day of recruitment are presented here.

Problems were recorded using the African Palliative Care Association (APCA) African Palliative Outcome Scale(POS), a multidimensional instrument designed[23] and validated in Africa[24]. A demographic questionnaire was also completed. The date and results of CD4 count and World Health Organization HIV stage were abstracted from patient records.

Recruitment and data collection was conducted by clinic health care workers, who received training and regular two-weekly support visits from research assistants. The questionnaires were translated and back-translated by experts into Swahili, Luo, Luganda and Runyakitara. The African POS had previously been validated in English and Luganda, among other languages[24]. The Swahili, Luo and Runyakitara translations were new. All translations were piloted with patients before use. Data were double-entered into a predesigned EpiData 3.1 database with validation checks, and exported to Stata 10.0 for analysis.

The POS consists of ten items addressing physical and psychological symptoms, spiritual, practical and emotional concerns, and psychosocial needs[24]. Seven items are completed by the patient and three by a family carer, but in this study it was anticipated that few patients would have a carer present and so only the patient-report items were analysed. POS scores were reversed where necessary so that 0 always represented the best response (no problem) and 5 the worst. Independent variables were gender; age group (18–28, 29–35, 36–59); country; physical function (measured with the ECOG scale[25], converted to 0 = best, 1 = middle, 2–4 = worst); education (no formal education, primary, secondary, diploma/degree); wealth quintile (calculated using the Demographic Health Surveys Wealth Index method[26]); CD4 count (0–100, 101–200, 201–350 and 351+); and time since HIV diagnosis (0 days, 1–2 days, 3–7 and 8–14 days). Patients were defined as ART eligible if they had a CD4 of 350 or below, or a WHO score 3 or 4, following the 2010 WHO guidelines for ART initiation[27], although when the data were collected in 2008 Kenya and Uganda had not yet adopted the eligibility threshold of 350.

### Analysis plan

The study outcomes are the three factors which have been identified underpinning the seven patient-completed items of the POS: physical/psychological wellbeing (questions 1, 2 and 3), interpersonal wellbeing (questions 4 and 7), and existential wellbeing(questions 5 and 6)[28]. These factors were generated by summing the scores for the appropriate questions. The ranges of the interpersonal and existential outcomes were 0–10, and of the physical/psychological outcome was 0–15, but preliminary analysis showed that no participants scored above 12.

For multivariate analysis each outcome was divided into three categories, coded ‘mild problems’ (0–4), ‘medium problems’ (5–7) and ‘severe problems’ (8–10 or 8–12). All independent variables were analysed as either ordinal (wealth quintile, education, physical function, age group, CD4 count, time since diagnosis) or dichotomous (gender, country, ART eligibility). The independent variables were compared with each outcome using chi square tests and non-parametric tests for trend, before being combined in a multivariate model using ordinal logistic regression.

## Results

Response rate was over 99%. Participants were 270 women (61.6%) and 168 men, aged 18–59 (mean 32.9); a little over half (56.4%, n = 247) were recruited in Kenya (Table 1). Age distribution was 20.3% aged 18–25, 63.0% aged 26–40 and 16.7% aged 41–59. The majority (69.9%, n = 306) were physically fully active (ECOG score 0), with 23.3% (n = 102) scoring 1 and 6.8%(n = 30) scoring more than 1. In terms of education, 19(4.3%) had none, 229(52.3%) primary education, 147(33.6%) secondary and 43(9.8%) a diploma or higher qualification. Six facilities recorded WHO stage for their 196 patients, with 26% Stage I, 36% Stage II, 31% Stage III and 7% Stage IV. A CD4 count was recorded for 303 participants (69.2%).

**Table 1:** Demographic characteristics of the newly-diagnosed participants by country.

Category	Subcategory	Kenya	Uganda
N		247	191
Gender, number female (%)		157 (63.6)	113 (59.2)
Age (mean, SD)		32.8 (8.7)	33.0 (8.3)
Age groups (%)	18–28	49 (19.8)	40 (20.9)
	29–35	159 (64.4)	117 (61.3)
	36–59	39 (15.8)	34 (17.8)
Education (%)	None formal	7 (2.8)	12 (6.3)
	Primary	141 (57.1)	88 (46.4)
	Secondary	78 (31.6)	69 (35.5)
	Diploma/higher	21 (8.5)	22 (11.5)
Physical function (%)	Best	180 (72.9)	126 (66.0)
	Middle	55 (22.3)	47 (24.6)
	Worst	12 (4.9)	18 (9.4)
Days since diagnosis (%)	0	29 (11.7)	79 (41.4)
	1–2	53 (21.5)	45 (23.6)
	3–7	102 (41.3)	35 (18.3)
	8–14	63 (25.5)	32 (16.8)
Wealth quintiles (%)	Poorest	63 (25.5)	25 (13.1)
	Second	51 (20.7)	37 (19.4)
	Middle	40 (16.2)	47 (24.6)
	Fourth	43 (17.4)	46 (24.1)
	Wealthiest	50 (20.2)	36 (18.9)
Has a recorded CD4 count (%)		222 (89.9)	81 (42.4)
Baseline CD4 count (median, IQR)		249 (95–407)	326 (193–483)
Has record of WHO stage (%)		124 (50.2)	(74 (38.7)
WHO stage (%)	I	32 (25.8)	20 (27.0)
	II	34 (27.4)	37 (50.0)
	III	47 (37.9)	15 (20.3)
	IV	11 (8.9)	2 (2.7)
Has ART eligibility score (%)		227 (91.9)	92 (48.2)
ART eligible (%)		162 (71.4)	56 (60.9)

For all seven items, the majority of participants reported problems (Table 2). The items with the highest scores were those measuring need for help and advice, and difficulty sharing feelings. Almost half of participants (47.3%) reported receiving no help or advice in the previous three days and almost a third (32.0%) had not been able to tell anyone how they were feeling. Further, 19.2% reported a peace score of 4 or 5, indicating a severe problem, 10.7% reported severe worry, 10.0% had a severe problem finding life worthwhile, 6.2% had severe pain and 3.2% severe physical symptoms other than pain.

**Table 2:** Prevalence and severity of multidimensional problems within 14 days of HIV diagnosis.

POS item	% of individuals scoring (n = 438)					
Over the past three days	0 (no problem)	1	2	3	4	5 (worst possible)
1. Please rate your pain	33.1	22.6	24.9	13.2	5.3	0.9
2. Have any other symptoms been affecting how you feel?	34.3	28.5	22.8	11.2	2.1	1.1
3. Have you been feeling worried about your illness?	31.5	22.4	26.0	9.4	5.7	5.0
4. Have you been able to share how you are feeling with your family or friends?	11.6	7.1	12.8	15.5	21.0	32.0
5. Have you felt that life was worthwhile?	42.7	21.2	14.8	11.2	5.0	5.0
6. Have you felt at peace?	24.4	21.7	23.1	11.6	8.0	11.2
7. Have you had enough help and advice for your family to plan for the future?	8.7	6.9	7.8	12.1	17.4	47.3

Reflecting the distributions of the individual items, physical/psychological problems were skewed to the left (45% mild problems, 43% moderate, 11% severe), as were existential problems (58% mild, 35% moderate, 7% severe). Interpersonal problems were skewed to the right (11% mild problems, 42% moderate, 46% severe).

In bivariate analysis, physical/psychological problems were associated with lower CD4 count, impaired physical function, poverty and less education at p<0.1. The presence or absence of a CD4 count was not associated with any of the three outcomes. Interpersonal problems were more common for those with better physical function, women, and individuals living in Uganda. Interpersonal problems were also associated with wealth quintile but in no clear direction. Existential problems were associated with residence in Uganda, impaired physical function, and younger age. ART eligibility was not associated with any outcome. A missing value for ART eligibility was associated with interpersonal problems, with country and with wealth quintile, so this variable was not included in the multivariate models.

Table 3 shows the results of multivariate models of each outcome. Physical/psychological problems were associated with impaired physical function (OR = 3.22, 95% CI 2.32–4.48) and to a lesser extent with poverty (OR = 0.85, 95% CI 0.74–0.98). Interpersonal problems were more common for women (OR = 1.51, 95% CI 1.03–2.24), and less common for people with more education (OR = 0.71, 95% CI 0.54–0.93) or impaired physical function (OR = 0.50, 95% CI 0.37–0.68). A longer time since diagnosis was also associated with fewer interpersonal problems (OR = 0.78, 95% CI 0.65–0.94). Existential problems were more common in Uganda than Kenya (OR = 2.40, 95% CI 1.58–3.63) and were also associated with impaired physical function (OR = 1.54, 95% CI 1.14–2.08), poverty (OR = 0.82, 05% CI 0.70–0.95) and weakly with younger age (OR = 0.80, 95% CI 0.63–1.02).

**Table 3:** Results of ordinal logistic regression models.

Risk factor (reference group)	Physical/psychological problems		Interpersonal problems		Existential problems	
	OR (95% CI)	p	OR (95% CI)	p	OR (95% CI)	p
Gender (male)	1.29 (0.87–1.92)	0.203	1.51 (1.03–2.24)	0.037	1.19 (0.79–1.79)	0.404
Age (18–28)	1.20 (0.95–1.52)	0.120	0.90 (0.72–1.14)	0.396	0.80 (0.63–1.02)	0.075
Education (none formal)	0.86 (0.65–1.13)	0.271	0.71 (0.54–0.93)	0.014	1.14 (0.86–1.52)	0.353
Wealth quintile (poorest)	0.85 (0.74–0.98)	0.027	0.92 (0.80–1.06)	0.242	0.82 (0.70–0.95)	0.009
Physical function (best)	3.22 (2.32–4.48)	<0.001	0.50 (0.37–0.68)	<0.001	1.54 (1.14–2.08)	0.005
Country (Kenya)	1.13 (0.76–1.68)	0.530	1.10 (0.75–1.63)	0.619	2.40 (1.58–3.63)	<0.001
Time since diagnosis (0 days)	1.03 (0.86–1.24)	0.709	0.78 (0.65–0.94)	0.008	1.03 (0.85–1.24)	0.773

## Discussion

Physical, psychological, existential and interpersonal problems are highly prevalent and severe in the first two weeks after HIV diagnosis. These problems are reported by patients to be barriers to retention in care[5], [6]. The sample consists of young adults, mainly women, as is typical of HIV clinic patients. None of the outcomes was associated with CD4 count, showing that problems can occur at any CD4 level.

The area of existential wellbeing is difficult to define and measure[29], but it is important to patients. Existential and spiritual concerns following HIV diagnosis are evident in qualitative studies[30], and for patients with advanced HIV in South Africa and Uganda, meaning in life is a higher priority than physical comfort or activity[14]. The question ‘are you/have you felt at peace?’ used in this study is an independently validated measure[24], [31]. Existential problems as measured by the POS are more severe among Ugandan than Kenyan participants. Possible causes of this effect include difference in care provision, questionnaire translation, cultural and health care context, or time passed since diagnosis (median four days in Kenya and one in Uganda). In Uganda almost two thirds of participants (63.9%) did not have a CD4 count on record a month after diagnosis, compared to only 13.8% in Kenya. This suggests that Uganda has either a lack of service availability or weaker care linkages. It is also possible that more CD4 tests were carried out and the problem was with study data collection.

Interpersonal problems – inability to access help and advice for the family or to share feelings – were reported most frequently. Those with more education have fewer interpersonal problems, which comprise lack of help/advice and inability to share feelings. In many cases these problems should be managed with counselling, which is a required element of care for all newly diagnosed patients[32]. The results suggest that lack of education limits the benefits of counselling for the patient. This could be caused by communication difficulties (low literacy, perhaps limited English), uncertainty what questions to ask, or reluctance to challenge health workers perceived to have authority. Interpersonal problems are more common for women, which is consistent with a multi-centre HIV study in 13 European countries which found that women received less emotional support than men and were also less likely to benefit from it[33].

Physical/psychological and existential problems are exacerbated by physical restriction and poverty, but those with impaired physical function actually report fewer interpersonal problems. The association may be affected by status disclosure. Physically active people with more resources may be more capable of concealing their HIV status, and non-disclosure could limit their ability to share their feelings and impede their access to help. There is evidence that people with more advanced disease are more likely to disclose their status and that disclosure is motivated by the need for material support[34], [35].

### Strengths and limitations

The study benefits from a sample recruited very soon after diagnosis and assessed using a validated, patient-reported instrument. This study includes a more representative sample than previous research in this area[15], [19] but its application to other contexts is limited. The clinics were purposively selected and are all in urban areas. Many patients were first tested for HIV at VCT services and referred to the clinic for care, which explains why only 25% of patients were seen on the day of diagnosis. Consecutive sampling can lead to selection bias if couples or friends with similar characteristics attend the clinic together. On the positive side, refusal was very low (<1%) and recall bias was minimised by data collection very soon after HIV diagnosis and limiting recall to a three-day period.

Risk factors are limited to one clinical and six demographic characteristics. This is reasonable because very little reliable information is available for this important patient group. CD4 count and WHO stage were both missing for large numbers of patients, especially in Uganda.

The African POS validation was conducted in palliative care facilities, not HIV clinics, and compared to the present study the patients had poorer physical function (only 11% scored 0 on the ECOG scale) and probably more advanced HIV, although CD4 count and viral load were not reported. However, the POS was designed for use at any stage of HIV infection, not only the end of life, and two of the validation facilities accepted referrals from the point of diagnosis. Only two of the five translations used in this study had been validated. The three new versions (Swahili, Luo and Runyakitara) were translated and back-translated according to best practice. The differences in existential well-being between Kenya and Uganda may be attributable to difficulties in translating this culturally variable concept.

The study has no HIV negative control group and so it does not identify the proportion of patient burden attributable to the physiological effects of HIV infection, to the psychological effects of HIV diagnosis, to indirect effects such as reduced income from illness, or to unrelated causes. From a public health perspective, the key finding is that multidimensional problems are a severe burden to newly diagnosed patients, and therefore they require effective care to improve wellbeing and aid retention whether the problems are related to HIV status directly, indirectly or not at all. Equally, the risk factors of poverty, physical restriction and limited education may be associated with multidimensional problems in the HIV negative population as well, but this would not affect the findings or conclusions of the study.

The odds ratios report the effect on the outcome of increasing the independent variable by one. For binary variables such as country, this represents the entirety of measured variation, whereas for ordinal variables such as education and wealth quintile it represents only a fraction, which limits the strength of associations for ordinal variables. Further details of problems such as duration, frequency and effect on daily living were not collected. Marital status, number of children, and HIV disclosure status were also not collected, and these demographic variables may have affected the outcomes.

## Conclusions

Clinical recommendations following from these findings are that newly diagnosed outpatients should receive assessment, screening and care for physical symptoms and psychological, spiritual and social problems within days of HIV diagnosis. Effective interventions must be developed and evaluated. A randomised controlled trial of the effectiveness of nurse training on multidimensional patient outcomes is in progress in Kenya and South Africa. The findings from this trial may be useful in the development of diagnosis-specific care support.

Clinical staff must be aware of the possibility of communication difficulties, and ensure patients have the opportunity to get the help and advice they seek. Patients need to be equipped with the means to overcome barriers to continued care, whether financial, structural, emotional or psychological. Those who see no benefit from care initially may be unlikely to return[36]. Future research should investigate whether outcomes improve over time from diagnosis after engagement with HIV services and ART initiation.
